# Trends in Hospital Admissions for Pertussis Infection: A Nationwide Retrospective Observational Study in Italy, 2002–2016

**DOI:** 10.3390/ijerph16224531

**Published:** 2019-11-15

**Authors:** Fabiana Fiasca, Giovanni Gabutti, Antonella Mattei

**Affiliations:** 1 Department of Life, Health and Environmental Sciences, University of L’Aquila, AQ 67100 L’Aquila, Italy; fabiana.fiasca@alice.it; 2 Department of Medical Sciences, University of Ferrara, FE 44121 Ferrara, Italy; giovanni.gabutti@unife.it

**Keywords:** pertussis, infection, hospitalization, vaccine, trend, booster doses, pregnancy, infants, adolescents, adults

## Abstract

*Background*: Pertussis is a highly contagious infectious disease which continues to be an important public-health issue despite the high immunization coverage rates achieved. However, evidence of increased circulation of pertussis among adolescents and adults due to waning immunity and atypical clinical manifestations seem to be the main reasons for its resurgence. The aim of this study was the analysis of the epidemiological trend for pertussis-related hospitalizations in Italy, in relation with vaccination coverage and information from laboratory confirmed cases of pertussis. *Methods*: A retrospective observational study investigating hospitalizations for pertussis from 2002 to 2016 in Italy was conducted. Frequencies and rates of hospitalization were analyzed and hospitalization data were compared with a series of already published laboratory confirmed data. *Results*: This study highlighted a rising trend for pertussis hospitalizations in Italy since 2008. Infants aged <1 year showed the highest frequencies (63.39%) and average rates (74.60 × 100000 infants) of hospitalization despite an extremely high vaccination coverage (95.89%). An increasing trend of hospitalization frequency emerged for the age group with levels of IgG antibodies to pertussis toxin compatible with pertussis infection within the last year (20–29 years old age group). *Conclusions*: The rising trend for pertussis hospitalizations and the greater involvement of infants aged <1 year require an integrated approach, including the implementation of booster doses administration in adolescence and adulthood, the vaccination of pregnant women and the *cocoon* strategy.

## 1. Background

Pertussis is a highly contagious infective disease mainly caused by the Gram-negative bacterium *Bordetella pertussis* (*B. pertussis*) [1].

Nevertheless, it is a vaccine-preventable disease: in Italy, the recommendation of a whole-cell vaccine (wP) since 1962, then replaced by acellular anti-pertussis (aP) vaccines in 1995, markedly reduced the frequency of the disease [2]. 

The aP vaccines are effective in preventing the clinical manifestations of pertussis, but it is still not clear whether they are unable to prevent colonization by *B. pertussis* and thus, impact the risk of transmission from a colonized subject to a healthy subject [3].

In addition, immunity to pertussis, whether conferred by vaccination or by natural infection, wanes over time and the clinical manifestations of the disease become less severe with age [1].

Adolescents and adults are now the main reservoir for the bacteria, in contrast to the pre-vaccination era: therefore, also due to the atypical clinical characteristics of pertussis in these subjects, they may become a significant source of infection for nonvaccinated or partially immunized newborns, in which the disease might be highly severe [4,5,6]. 

The epidemiological role of adolescents and adults in pertussis transmission and the waning immunity explain the introduction of booster vaccine doses into the Italian vaccination calendar for preschool children and for adolescents and adults (aged 19 years or more), to be repeated every 10 years [7]. 

Moreover, the immune pressure from vaccinations was thought to be responsible for the emergence of vaccine escape mutants of *B. pertussis* [8].

All the above-mentioned topics were considered as possible causes of pertussis resurgence, along with detection bias due to increasing public awareness of the disease [9]. Official data, in fact, indicated a resurgence of pertussis in Europe despite high vaccination coverage, with a peak of incidence in 2012 [10]. During this year, the notification rate of pertussis cases was more than twice as high as in the previous year and the overall rate of confirmed cases was more than 2.5 times higher than in 2003–2007. However, this increase was not uniform across the European countries: the highest number of cases in 2012 was reported in the Netherlands and in the United Kingdom, where it was ten times higher than in 2011. Significant increases of pertussis cases were reported for other countries, such as Austria, Czech Republic, Denmark, Ireland, Latvia, Lithuania, Poland and Portugal [10].

As regards the Italian situation, we are observing an alarming increase in cases of illness, doubled between 2008 (*n* = 345) and 2009 (*n* = 638), and subsequent hospitalization rate, probably also due to a reduction in vaccination coverage. In Italy, in fact, a strong wave of skepticism led to a general decline in having vaccinations [11].

To analyze the epidemiological impact of vaccination coverage on pertussis infection in Italy, a nationwide study evaluating frequencies and hospitalization rates, also in relation with information from laboratory confirmed cases of pertussis, was conducted.

## 2. Methods

A retrospective observational study investigating hospitalizations for pertussis from 1 January 2002 to 31 December 2016 in Italy was carried out using the Hospital Discharge Database (HDD) as informational flow. The following codes of the International Classification of Diseases, ninth revision, Clinical Modification (ICD9-CM) system were searched: 033.0 (pertussis due to *B. pertussis*), 033.1 (pertussis due to *B. parapertussis*), 033.8 (pertussis due to other specified pathogens), 033.9 (pertussis due to unspecified pathogens), and 484.3 (pneumonia in pertussis). In this study, we included all admissions with at least one pertussis-related main or secondary discharge diagnosis.

The hospitalization rates × 100000 people were calculated using the Italian resident population provided by the Italian Institute of Statistics (ISTAT) during the period 2002–2016; surveillance data about vaccination coverage for pertussis at age 24 months during the study period were provided by the Ministry of Health database [12,13]. The coverage at each year was defined as the proportion of children born that year who received three or more doses of aP vaccine. 

Descriptive statistics were performed: the discrete and nominal variables (gender, age classes, nationality, geographical location, number of deaths, and the presence of concomitant chronic respiratory diseases) were expressed by frequencies and percentages; the continuous variables (age, length of hospital stay and costs related to admissions) were expressed as mean values and standard deviations (SDs).

To analyze frequencies and rates of hospitalization four age groups were considered: <1, 1–4, 5–14 and ≥15 years of life. 

To compare hospitalization data with a series of already published laboratory confirmed data, three age classes were analyzed: 20–29, 30–39, and ≥60 years of life [14]. These age groups were selected because they could be a reservoir of *B. pertussis* for transmission to infants [14]. 

The temporal trend was analyzed by the slope of the regression line. A linear regression analysis was performed to test the relationship between vaccination coverage at 24 months of life and hospitalization rates, also stratified for age classes. Scatter plots were used to display these relations. 

*p* < 0.05 was the criterion for statistical significance. A data analysis was performed using STATA/IC 15.1. 

Ethics Approval and Consent to Participate: Data provided by the Ministry of the Health did not contain any patient identifiers and was therefore completely anonymous. Hence, notification of the study to Ethics Committees was not applicable, nor was informed consent of patients required.

Availability of Data and Materials: Hospital discharge records are available at the National Archive of HDRs data, Ministry of Health, General Directorate of Healthcare Planning, VI Office. The datasets analysed during the current study are available from the corresponding author upon reasonable request.

## 3. Results

In the period 2002–2016, 9393 patients were hospitalized for pertussis in Italy, with an annual mean equal to 626 hospitalizations. Considering only the cases with pertussis listed as the first diagnosis, the number of hospitalizations was 7137 and the code 033.9 (pertussis due to unspecified pathogens) resulted to be prevalent (57.48%, 4102/7137) (code 033.0 “pertussis due to *B. pertussis*”: 34.87%, 2489/7137; code 033.1 “pertussis due to *B. parapertussis*”: 3.56%, 254/7137; code 033.8 “pertussis due to other specified pathogens”: 2.31%, 165/7137; code 484.3 “pneumonia in pertussis”: 1.78%, 127/7137) (data not shown).

The characteristics of the study population are summarized in Table 1. In total, 52.60% (4941/9393) were female. The mean age was 6.32 years (SD = ±20.57) and the majority of cases occurred in the <1 year age group (5954/9393, 63.39%). A total 5.87% (551/9393) of cases were immigrants and slightly more than half of the hospitalizations were registered in the South and Islands (4928/9393, 52.46%). In total, 34 deaths were reported, with a case fatality rate equal to 0.36 (34/9393). In eight cases (8/34, 23.53%) the diagnosis of pertussis was the principal diagnosis and only one of those (1/8, 12.50%) reported the code relative to *B. pertussis* infection (data not shown). When pertussis was reported as one of the secondary diagnoses (26/34, 76.47%), the prevalent principal diagnosis code (8/26, 30.77%) was 518 (“Other diseases of lung”). A total 3.97% (373/9393) of admissions presented concomitant chronic respiratory diseases (mainly asthma; data not shown) and the average length of stay was equal to 6.59 days (SD = ±7.81). Total hospital charges for the admissions for pertussis in the overall period were approximately €17 million, with a mean equal to €1806 per hospitalization (SD = ±2236). 

**Figure 1:**
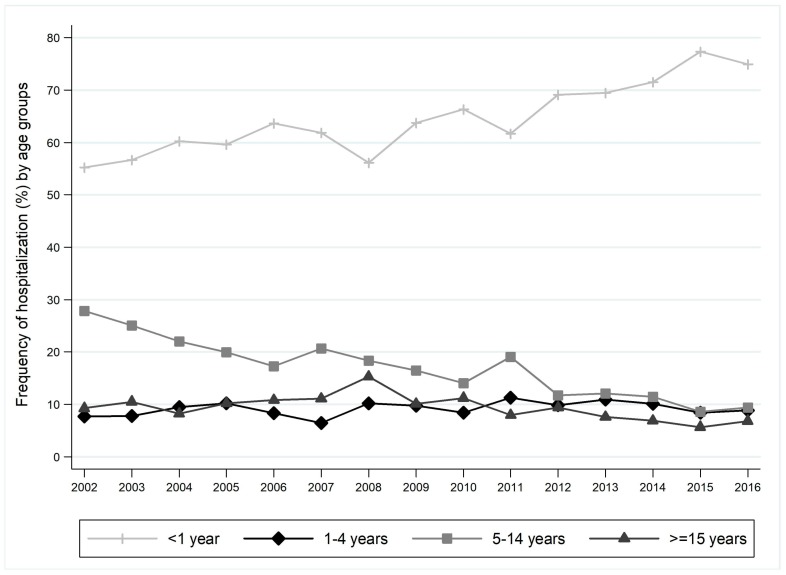
Frequency of hospitalization for pertussis in Italy stratified by age groups (2002–2016 time-frame). Trend test. <1 year: β coefficient = 1.37, *p* < 0.001; 1–4 years: β coefficient = 0.11; *p* = 0.178; 5–14 years: β coefficient = −1.20; *p* < 0.001; ≥15 years: β coefficient = −0.28; *p* = 0.045.

The frequency of hospitalization stratified by age groups showed an increase from 55.26% in 2002 to 74.92% in 2016 in the <1 year age class (Figure 1) with a significant trend (β = 1.37; *p* < 0.001). A significant decreasing trend emerged for the age classes 5–14 years (β = −1.20; *p* < 0.001) and ≥15 years (β = −0.28; *p* = 0.045).

**Figure 2:**
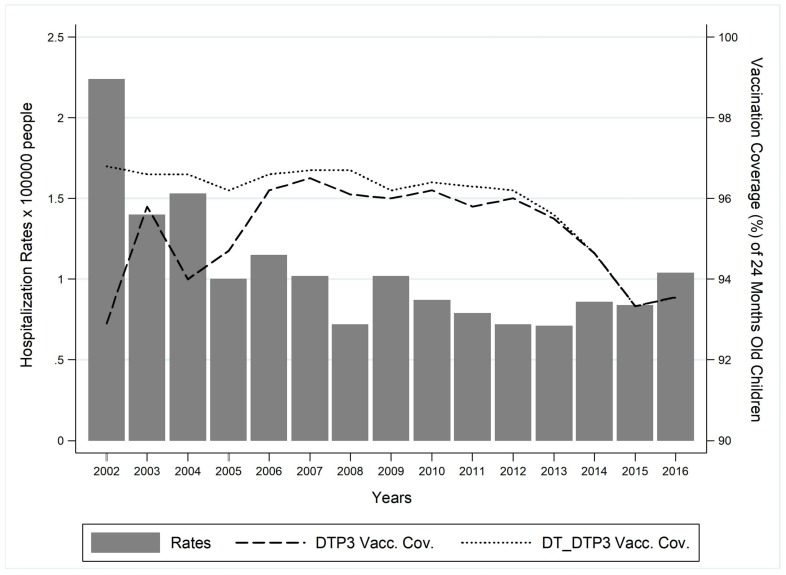
Temporal trend of hospitalization rates and vaccination coverage of 24 months old children for pertussis in Italy (2002−2016 time-frame). Trend test. Hospitalization rates: β coefficient = −0.06; *p* = 0.003. DTP3 coverage: β coefficient = −0.20, *p* = 0.785; DT-DTP3: β coefficient = −0.20, *p* < 0.001.

Analysing the hospitalization rates × 100000 people (Figure 2), a minimum value was highlighted in 2008 and the average hospitalization rate resulted in 1.06 × 100000 people in the study period. The overall temporal trend was decreasing (β = −0.06; *p* = 0.003) but after 2008, the trend started to rise up again (β = 0.01, *p* = 0.540).

When the trend of hospitalization rates was compared to vaccination coverage of 24 months old children for pertussis (Figure 2), a significant decreasing trend of the adhesion to vaccination after 2008 was shown (DTP3: β = −0.42, *p* = 0.002; DT-DTP3: β = −0.42, *p* = 0.001), mainly from 2011 onwards. Nevertheless, at national level, pertussis vaccination coverage was high, the average being slightly more than 95% during the study period (average vaccination coverage: DTP3 = 95.15%; DT-DTP3 = 95.89%), although the coverage levels dropped below this threshold after 2013.

**Figure 3:**
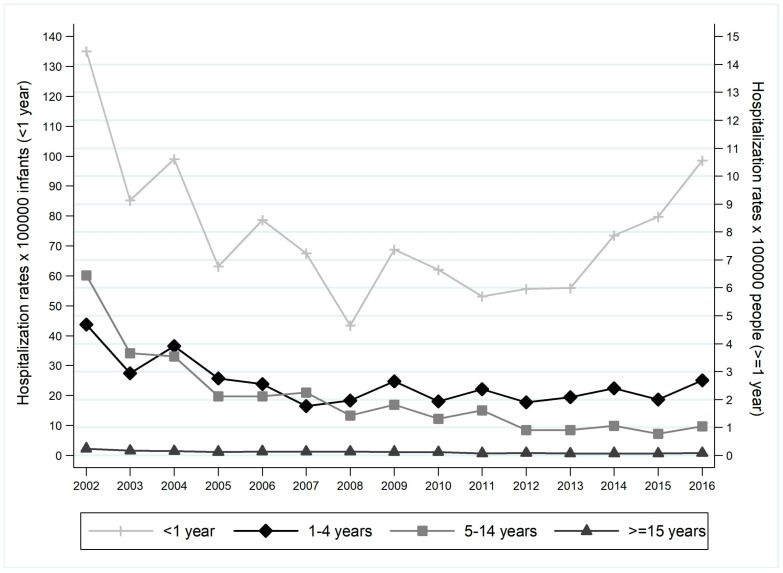
Hospitalization rates × 100000 people by age groups. Trend test. <1 year: β coefficient = −1.87, *p* = 0.186; 1–4 years: β coefficient = −0.11, *p* = 0.015; 5–14 years: β coefficient = −0.28, *p* < 0.001; β coefficient = −0.01, *p* < 0.001.

Analysing the hospitalization rates by age groups (Figure 3), a significant increasing trend after 2008 emerged for the <1 year age class (β = 4.66; *p* = 0.016), which presented the highest hospitalization rates (average hospitalization rate: 74.60 × 100000 infants). The 5–14 years and ≥15 years age groups, on the other hand, showed a significant decreasing trend after 2008 (β = −0,10, *p* = 0.018; β = −0.01, *p* = 0.008, respectively), and with average hospitalization rates equal to 2.06 × 100000 people and 0.12 × 100000 people in the study time frame. 

**Figure 4:**
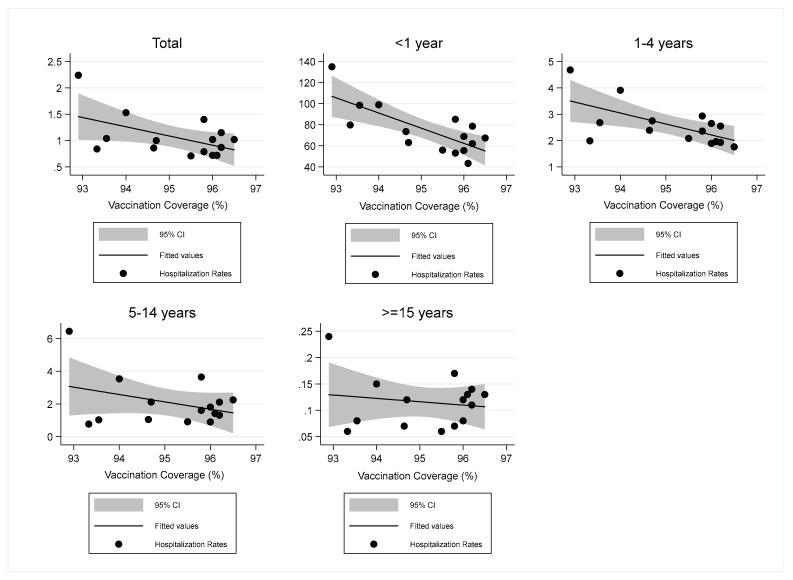
Scatter-plots and regression lines of pertussis hospitalization rates vs. vaccination coverage of 24 months old children in Italy (2002–2016 years). Linear regression analysis. Total: regression coefficient (95% CI) = −0.18 (−0.35–−0.01); <1 year: regression coefficient (95% CI) = −14.40 (−22.13–−6.68); 1–4 years: regression coefficient (95% CI) = −0.42 (−0.73–−0.10); 5–14 years: regression coefficient (95% CI) = −0.45 (−1.15–0.25); ≥15 years: regression coefficient (95% CI) = −0.01 (−0.031–0.018).

The relationship between overall pertussis hospitalization rates and vaccine coverage was quite linear (β = −0.18; 95% confidence interval (CI): −0.35–−0.01; *p* = 0.048), as shown in Figure 4. The linear regression analysis highlighted a significant reduction of overall hospitalization rates (*p* = 0.048), and hospitalization rates for <1 year (*p* = 0.001) and 1–4 years (*p* = 0.013) age groups for one unit of change in the vaccination coverage of 24 months old children, as shown in Table 2. Notably, the regression coefficient indicated that for every additional percentage point of vaccination coverage, the hospitalization rates decreased by an average of 14.40 × 100000 in the first year of life. Moreover, r squared (r^2^) indicated that 55.5% of the variance of hospitalization rates for this age class was explained by vaccination coverage. 

**Figure 5:**
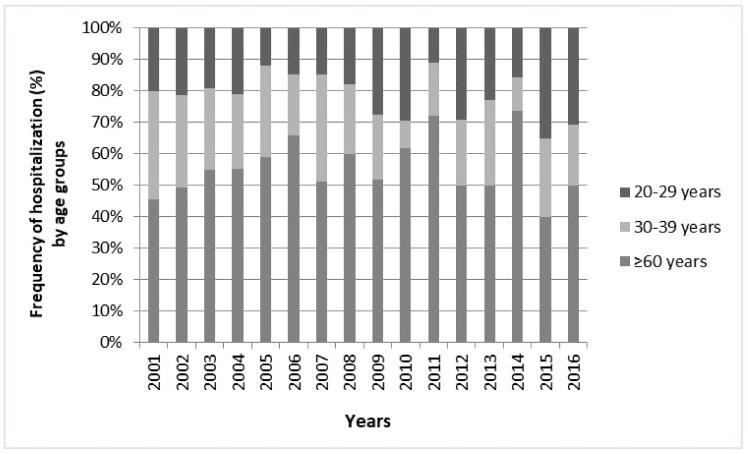
Frequency of hospitalization for pertussis for 20–29 years, 30–39 years and ≥60 years age classes in Italy (2002–2016). Temporal trend. 20–29 years: β coefficient = 0.81, *p* = 0.065; 30–39 years: β coefficient = −0.71, *p* = 0.086; ≥60 years: β coefficient = −0.11, *p* = 0.855.

To compare hospital discharge data with information from a series of already published laboratory confirmed pertussis cases, three age classes were analyzed: 20–29 years, 30–39 years, and ≥60 years (Figure 5). According to Palazzo et al., the highest percentage of subjects with levels of immunoglobulin G (IgG) antibodies to pertussis toxin (PT-IgG) compatible with pertussis infection within the last year (≥100 IU mL^−1^) was in the 20–29 years age group (7.1%, 17/239) [14]. The temporal trend of the frequency of hospitalization for pertussis for this age class was increasing in the study period (β = 0.81; *p* = 0.065). However, the trend was decreasing for 30–39 years and ≥60 years age classes (β = −0.71, *p* = 0.086; β = −0.11; *p* = 0.855, respectively). 

## 4. Discussion

Our analysis confirms pertussis as a relevant public health concern in Italy and highlighted a significant negative relation between hospitalization rates, especially for children aged <1 year and vaccination coverage. One percent more vaccination coverage with the primary immunization course with aP should be sufficient to reach a reduction of hospitalization rates in the first year of life equal to 14.40 × 100000 infants. 

The analysis of hospitalizations shows an increasing trend for pertussis in Italy, mainly after 2011, despite high coverage with pertussis-containing vaccines. This increasing trend was also observed in other European countries, where the number of cases reported to the World Health Organization showed a notable increase in 2012 [15,16].

Several contributing factors could affect this apparent resurgence of pertussis as waning immunity, pathogen adaptation, and improved diagnostics [17].

Comparing hospital discharge data with information from a series of already published laboratory confirmed pertussis cases, an increasing trend of hospitalization frequency emerged for the age group (20–29 years old) with PT-IgG levels compatible with pertussis infection within the last year (≥100 IU mL^−1^) [14]. This result suggested that the 20–29 age groups appear to be more exposed to pertussis and could be a good indicator of an increase in circulation of *B. pertussis* in the country.

Hence, there is a need to implement the administration of booster doses in adolescence and adulthood. 

The duration of protection and thus, the waning immunity, could be also influenced by the significant changes in *B. pertussis* populations, observed after the introduction of vaccinations [17]. 

Moreover, it was demonstrated that the protection evoked by all of the pertussis vaccines tends to wane with time, based also on vaccination schedule and the type of vaccine [10]. The aP vaccine, in fact, seems to provide shorter-lasting protection than the wP vaccine [10].

Regarding the diagnostic issue, our data highlight that the code 033.9, related to pertussis due to unspecified pathogens, resulted in the most prevalence (57.48%) in Italy: therefore, more efforts should be made to implement the etiological diagnosis in order to reduce the under-notification of the disease.

Recent studies indicated a correlation between pertussis and Chronic Obstructive Pulmonary Disease [18]. Pertussis with concomitant respiratory diseases represented almost 4% of hospitalizations in the study period. Moreover, the finding of more hospitalizations in South and Islands could be partly explained by the observation of the circulation of a hypervirulent strain of *B. pertussis*, also found in vaccinated people, among the population of this part of Italy [19].

Analyzing the relationship between hospitalization rates and vaccination coverage for pertussis, a significant linear negative relation was discovered: this suggests that maintaining a high vaccination coverage (>96%) is essential to control the disease. Indeed, 55.5% of the variability of hospitalization rates for children aged <1 year was attributable to vaccination coverage. The observed decreasing coverage for anti-pertussis vaccine, as well as for other vaccinations, since 2008, reflected an international trend recorded in the past few years and it was mainly due to what is known as “vaccine hesitancy” [20]. It was defined as “a delay in acceptance or refusal of vaccines despite availability of vaccination services” [21]. Missing or inadequate communication could contribute to vaccine hesitancy and negatively influence vaccination uptake. Better communication in the field of vaccination in Italy will be therefore essential to avoid lowering the vaccine coverage threshold to below 95% of the population, as, according to WHO, this is the minimum population coverage value needed to prevent vaccine-preventable diseases [22]. Greater frequencies and rates of admission for the <1 year age group, according to previous Italian studies [6,23], were also consistent with regional data from laboratory confirmed pertussis cases: 60% of *B. pertussis*-positive samples were of children less than 1 year of age [19].

Thus, infants appeared to be exposed to pertussis before developing vaccine-induced immunity, being the vaccination coverage at 24 months old extremely high in the study period. It was demonstrated that hospitalizations due to pertussis disproportionately affect children less than three months of age [24]. 

This could be explained partly by the fact that at least two doses of pertussis-containing vaccine are necessary to adequately protect children, partly because the first dose is not given until three months of age in Italy [24].

Thus, it is important to protect infants from pertussis until they are old enough to be vaccinated. To this end, a winning strategy has proven to be the maternal immunization: it provides transient immunity to the newborn by the transplacental transfer of maternal IgG antibodies or via breast milk of maternal IgA antibody to pertussis toxin [25,26]. 

The maternal immunization has been recommended as a strategy in many resource-rich countries, including the USA since 2011, the UK since 2012, and Australia since 2015 [24].

It is important to remember that accordingly to the most recently published data, no increased risk of adverse events among women who received aP vaccine during pregnancy or among their infants has been registered [27].

In addition to vaccination of pregnant woman, the *cocoon* strategy, which consists of vaccination of the family members and close contacts of the neonate, was also proposed to reduce the risk of severe pertussis infection in younger infants [10,28,29]. 

In fact, when a source of pertussis infection could be identified, parents, siblings, or other infant caregivers were typically responsible for transmitting the disease to vulnerable infants [30].

The administration of an aP dose to close contacts could significantly reduce the risk of pertussis in the first months of life [31].

Limitations of the study. Using the hospital discharge database as informational flow, some diagnoses could be excluded [32,33,34]. 

In addition, an underestimation of pertussis related hospitalizations in adolescent, young adults and adults could take place mainly related to the atypical clinical characteristics of cases and the lack of laboratory confirmation [6]. 

Lastly, the vaccination status of each case included in the analysis was not considered, as these data were not available.

## 5. Conclusions

Infants aged <1 year showed the highest frequencies and rates of hospitalization for pertussis in Italy. An increase of 1% of vaccination coverage with the primary immunization course should be sufficient for decreasing hospitalization rates of 14.40 × 100000 infants, which is the decrease of vaccination coverage responsible of more than half of infant hospitalizations. To improve the protection of this sensitive age group, prompt and effective measures should be also implemented for the administration of booster doses in adolescence and adulthood, for the vaccination of pregnant woman and for the cocoon strategy. 

## Figures and Tables

**Table 1:** Characteristics of the patients hospitalized for pertussis in 2002–2016, in Italy.

Investigated Variables	*N* = 9393
Gender, *n* (%)	
Male	4452 (47.40)
Female	4941 (52.60)
Age (years), mean ± SD	6.32 ± 20.57
Age classes, *n* (%)	
<1 year	5954 (63.39)
1–4 years	841 (8.95)
5–14 years	1718 (18.29)
≥15 years	880 (9.37)
Nationality, *n* (%)	
Italian	8842 (94.13)
Immigrant	551 (5.87)
Geographical location, *n* (%)	
North	2630 (28.00)
Centre	1835 (19.54)
South and Islands	4928 (52.46)
Deaths, *n* (%)	
No	9359 (99.64)
Yes	34 (0.36)
Concomitant chronic respiratory diseases, *n* (%)	
No	9020 (96.03)
Yes	373 (3.97)
Lenght of hospital stay (days), mean ± SD	6.59 ± 7.81
Hospital charge (€), mean ± SD	1806 ± 2236

**Table 2:** Linear regression analysis between hospitalization rates × 100000 people and vaccination coverage of 24 months old children in Italy (2002–2016).

Hospitalization Rates	Vaccination Coverage of 24 Months Old Children		
	Regression Coefficient	r^2^	*p*-Value
Total	−0.18	0.269	0.048
<1 years	−14.40	0.555	0.001
1–4 years	−0.42	0.387	0.013
5–14 years	−0.45	0.129	0.189
≥15 years	−0.01	0.024	0.581

## List of Abbreviations

aP: acellular anti-pertussis. *B. parapertussis: Bordetella parapertussis. B. pertussis: Bordetella pertussis*. CI: confidence interval. DT-DTP3 and DTP3: Diphtheria-Tetanus-Pertussis vaccine. HDD: Hospital Discharge Database. ICD9-CM: International Classification of Diseases, ninth revision, Clinical Modification system. IgA: immunoglobulin A. IgG: immunoglobulin G. IU: International Unit. PT-IgG: IgG antibodies to pertussis toxin. SD: standard deviation. UK: United Kingdom. USA: United States of America. wP: whole-cell vaccine.
